# Surgery

**Published:** 1898-03

**Authors:** W. M. Barclay


### SURGERY.

Dr. Calot, of Berck, is responsible for introducing anew the **treatment of Pott's disease of the spine by forcible reduction**—a method so opposed to the established canons of surgery, and suggested for a class of cases of such gravity, that its issue, as it shall be found to stand or fall, cannot fail to be of supreme importance and interest. In his original communication[5] he affirms, from an experience of 37 cases, that all children with Pott's curvature can be cured without deformity by forcibly correcting the curve as soon as it appears. In November last he contributed a paper to the discussion on this subject at the Clinical Society of London,[6] which was introduced by Messrs.

[5] *Gaz. d. Hôp.*, 1896, lxix. 1447; abstract in *Brit. M. J.*, 1897, i. Epitome, p. 18.

[6] *Brit. M. J.*, 1897, ii. 1501.

Tubby and Jones. He maintained that the difficulties and dangers of the correction were not greater in spinal disease than in disease of joints; and while admitting elements of risk from the anæsthetic, from paralysis, and from tubercular dissemination, he thought the results so far attained very satisfactory; and by dispensing with chloroform, and selecting the cases more carefully, he looked for still better in the future. He classified as unsuitable (1) children in an advanced condition of cachexia; (2) children who suffered from cough; (3) children with abscesses —except after treatment of the abscess by puncture and injection of iodoform, naphthol, etc., which was the only method he countenanced; (4) children with "fistulæ"—unless these could be first cured, a result he did not seem to anticipate; (5) persons over twenty or twenty-five years of age with deformity of long standing, *i.e.* seven or eight years, which resisted traction of from 50 to 80 kilogrammes (110 to 176 pounds). The cases which resisted the latter degree of force did so because of bony adhesions, which were situated either posteriorly between the laminæ and spines, or anteriorly between the bodies as well; and for these, and for all cases which resisted a traction of 100 kilogrammes (220 pounds), he was in favour of a preliminary osteotomy to separate the united laminæ or spines, and, after allowing a fortnight for the wound to heal, completing the reduction in the usual way. This procedure he expected would be sufficient even when there were anterior adhesions; but if it failed, he proposed dealing with these by a second osteotomy, reaching the bodies from the side and taking care to avoid entering the spinal canal.[1]

With regard to **results** after forcible reduction, he asserts that the spine undergoes consolidation just as it does when left to itself. But this bold and general assertion is scarcely justified by such details as are given. "He had radiographs which clearly showed that union took place in some cases by true ankylosis, when the deformity was slight or of medium degree;" but when the deformity was "very considerable," the only consolidation was by ankylosis between laminæ and articular processes, sometimes also between spines: and to make this efficient, it should involve the whole length of the projection, and even extend a little above and below it. Among thirteen recent cases, that is where the tubercular disease is still active, in four "spontaneous consolidation" occurred in from six to ten months, whereas seven were insufficiently consolidated: which means, according to the author, that an insufficient number of laminæ were ankylosed. If after waiting four or five months this condition was found, he recommended an operation to bring about the necessary extent of ankylosis, by apparently stripping periosteum off the united laminæ and applying it where it was desired to promote further ankylosis. The description of the author's method of producing and maintaining

[1] *Brit. M. J.*, 1897, ii. 1503.

reduction in ordinary cases occupies the rest of the paper. Dr. Rédard[1] and Mr. Tubby[2] have devised modifications which simplify the operation; and the latter, in conjunction with Mr. Jones, recommends the employment of a modified double Thomas's hip-splint with an adjustable head-piece in place of the cumbersome and risky plaster casing described by Dr. Calot. Willems,[3] who adopts Calot's method, advocates its being carried out without chloroform, mainly on account of the com pression of the chest during the manipulations, and also in the application of the plaster support, when, if danger threatens from respiratory embarrassment or failure, little can be done. The pain is not nearly so bad as might be expected, and is certainly not acute. Chloroform may, however, be necessary in older children and in adults.

This is the case for forcible and immediate reduction as it is put by Dr. Calot and his followers, and in support of it he cites an astonishingly large number of patients that have been subjected to the treatment. He speaks of nearly three hundred cases of his own, and of six hundred others reported to him from various quarters. If to these we add the sixty cases of Messrs. Tubby and Jones, and the fourteen of Mr. Murray,[4] in this country, we have a total of about one thousand cases. Only ten deaths are recorded. But this surprising record is rendered almost valueless by the absence, except in the rarest instances, of details that are essential in forming a judgment, where such could have been given; and by the extremely short time that must have elapsed since most of the cases were brought under treatment. We are not told, in most cases, the degree and extent of the curve; the stage of the disease; the lapse of time between treatment and report; the condition of the spine since reduction of its deformity; the condition found *post-mortem* in the fatal cases. To take these in order:

*Degree and extent of curve.*—From Dr. Calot's classification of the spinal deformity as "slight," "of medium degree," and "very considerable," and the fact that he only specifically mentions thirteen cases under the last head, we might perhaps infer that the majority of his cases were of the lesser degrees of severity. At the Clinical Society's discussion the President (Mr. Langton), in reference to Messrs. Tubby and Jones's cases—twenty-five in all,—remarked that "in some of the cases the deformity seemed so slight before operation that a cure would probably have resulted from prolonged rest in bed only."[5]

*Stage of the Disease.*—Whether, that is, the disease was active or quiescent when reduction was made, and its duration in months or years. In respect of the first half of the question, Calot records eight cases of paraplegia; Tubby and Jones, five cases;

[1] *Brit. M. J.*, 1897, ii. 1643. [2] *Practitioner*, 1898, lx. 31.

[3] *Semaine méd.*, July 28th, 1897; abstract in *Brit. M. J.*, 1897, ii. Epitome, p. 66.

[4] *Brit. M. J.*, 1897, ii. 1504. [5] *Ibid.*, 1501.

Murray, one case, and one of small psoas abscess. The paraplegia, it may be noted, was relieved in all these cases. As to duration of the disease, no indication is given of the proportion of early to long-standing cases.

*Lapse of time between treatment and report.*—Herein probably lies the weakest point of the whole argument. We have to rely entirely on deduction. Dr. Calot, at the time of his first communication on December 22nd, 1896, had carried out his method in thirty-seven cases; therefore the remaining two hundred and sixty of his cases must have occurred between that time and November 12th, 1897. This interval is 10½ months; and, allowing for the growth of a surgeon's reputation, we cannot be far wrong in assigning the larger proportion of the cases to the later months. Messrs. Tubby and Jones's cases extend over six months. Mr. Murray's experience dates from August 20th, 1897. No attempt is made to differentiate the cases, although the longest lapse of time possible would be far too short for any sound general deduction.

*Condition of spine after reduction.*—No evidence is forthcoming, except the four cases of Dr. Calot's, in which "spontaneous consolidation" was said to have occurred in from six to ten months, and his radiographs which clearly showed union by true ankylosis in *some* cases. A case mentioned by Mr. Noble Smith[1] is instructive in this connection. It was that of a boy in whom a curved spine had been straightened by the position he adopted of lying on his knees and elbows; in two years he was able to walk about, but a little later died of tubercular meningitis. The cavity in front of the spine had been filled when fresh with bony formation, but the new bone was so friable that it came away during preparation; so that in two and a half years no permanent repair had taken place. On the other hand, Calot, Tubby, Jonnesco,[2] all speak of recurrences of the deformity after reduction, requiring a second or third application of the method.

*Post-mortem appearances of the Spine.*—The failure to supply this information is inexplicable. Considering the gravity of the disease, and the serious responsibility that must rest with anyone who urges the adoption of a line of treatment that flies in the face of all experience and teaching, it might have been taken for granted that such important evidence as this would have been carefully scrutinised and reported. As it is, the record of these appearances in Mr. Murray's two cases[3] is the only one vouchsafed to us, and their evidence is not encouraging. In the first case the child died less than two months after reduction, of pneumonia (not tubercular): "There was not the slightest evidence of repair, but the gap formed . . . had resolved itself into a 'false joint' bounded by caseous and diseased tissue."

[1] *Brit. M. J.*, 1897, ii. 1644. [2] Quoted in *Ann. Surg.*, 1897, xxvi. 737.
[3] *Brit. M. J.*, 1897, ii. 1630.

There was no abscess, and no evidence of general tuberculosis. The second case died three months after operation, and the same entire absence of repair, or of filling up of the gap, was found, and the back was very "wobbly" at the seat of fracture. There was general tuberculosis.

The advantages claimed for this method by Messrs. Tubby and Jones are: (1) the greater ease of fixation of a straight than of a crooked spine; (2) the avoidance of profound alterations in the chest-wall leading to cramping and displacement of the abdominal and thoracic viscera, and more especially the heart and great vessels; and (3) æsthetic reasons. The first is hardly adequate as a reason for discarding safer methods: the moderate cases will improve under the old treatment, while the more pronounced and inveterate the curve the less favourable does the case become for forcible reduction. The same argument applies with even greater force to the second claim. The æsthetic argument cannot be allowed on its own merits. We have to deal, as Mr. Watson Cheyne said,[1] not with a deformity only, but also with a disease; and we cannot speak of a cure until the tuberculous bone disappears or becomes encapsuled, and bony ankylosis has resulted. Dr. Calot makes light of the risk of tubercular dissemination, and asserts that it is less to be dreaded after forcible correction of spinal curvature than after operations on the knee, and accounts for this difference by assuming that in the former case the segments of tuberculous bone are separated "without rupture of the trabeculæ," while the pressure in the posterior parts is borne on bony structures that are quite healthy. Yet Calot himself, and Tubby, and Murray, have recorded cases where tubercular disease has been apparently lighted up by this procedure; and Noble Smith[2] records three cases of a similar kind. Nevertheless, the freedom from this catastrophe has certainly been remarkable. Can the explanation be that enough time has not elapsed? Even if the slumbering foci of disease are disturbed, absolute fixation and rest of the part is maintained, and the subsequent condition is therefore as favourable as possible; if no general dissemination is caused at the time, the local re-infection may smoulder, and either die out again or gradually gain the mastery, and show its presence at some remoter period, locally or generally. Secondly, as to bony ankylosis: tubercular disease is not conducive to bone-formation around the infected area, and a large cavity filled with blood-clot and cheesy masses is not a state of things to encourage new development of bone, nor is the large cavity that results in these cases likely to be bridged over by bone. M. Ménard writes in the same sense.[3] "Besides the injury of no one knows how grave a character that this violent treatment may produce, there is no reason to believe that there can be a permanent cure obtained, for pathological specimens

[1] *Brit. M. J.*, 1897, ii. 1643. [2] *Loc. cit.*

[3] *Gaz. hebd. de Méd.*, 1897, N.S. ii. 505; abstract in *Am. J. M. Sc.*, 1897, cxiv. 721.

obtained at various stages of the disease show that no new growth of bone occurs to take the place of the bone destroyed by the disease, and there is no growth which can fill up the gap left where the vertebræ have been destroyed and give a sufficient solidity to the spinal column." He therefore concludes that this procedure is without foundation, is dangerous to the patient, and has neither given, nor on pathological grounds can be expected to give, results to prove its value.

It is significant that at the discussion[1] to which frequent allusion has been made, most of the responsible speakers were opposed to the innovation, or at least pleaded that it would be wiser as well as safer to await the result of the treatment in those cases that had already been submitted to it, till sufficient time had passed to warrant an estimation of its value, that is to say about two years hence. This seems to be the only reasonable conclusion in a matter of such importance, and where the evidence produced is so imperfect and premature.

In connection with this method of treatment, Mr. Noble Smith[2] has recently suggested that cases of lateral curvature of the spine might be benefited by forcible correction combined with the use of apparatus, and that without the risks attending the same procedure in Pott's disease. He reports a case which is in progress of treatment, and believes that he has effected a more rapid amelioration than he would have secured by means of his apparatus alone. He advocates only manual force in the corrections, and believes he can influence the rotation of the vertebræ. He bases his advocacy on his experience that whatever position can be obtained by apparatus can always be reckoned upon to become permanent in the course of time.

W. M. BARCLAY.

[1] *Brit. M. J.*, 1897, ii. 1501, 1642. [2] *Ibid.*, 1898, i. 76.

